# Manipulation of two regulatory genes for efficient production of chromomycins in *Streptomyces reseiscleroticus*

**DOI:** 10.1186/s13036-018-0103-x

**Published:** 2018-06-07

**Authors:** Lei Sun, Jia Zeng, Peiwu Cui, Wei Wang, Dayu Yu, Jixun Zhan

**Affiliations:** 1 Department of Biological Engineering, Utah State University, 4105 Old Main Hill, Logan, UT 84322-4105 USA; 2 TCM and Ethnomedicine Innovation & Development Laboratory, School of Pharmacy, Hunan University of Chinese Medicine, Changsha, Hunan 410208 China; 3 Hangzhou Viablife Biotech Co., Ltd., 1 Jingyi Road, Yuhang District, Hangzhou, Zhejiang 311113 China

**Keywords:** *Streptomyces reseiscleroticus*, Chromomycins, Biosynthesis, PadR-like transcription regulator, SARP regulator

## Abstract

**Background:**

Regulatory genes play critical roles in natural product biosynthetic pathways. Chromomycins are promising anticancer natural products from actinomycetes. This study is aimed to create an efficient strain for production of these molecules by manipulating the regulatory genes.

**Results:**

A putative but silent chromomycin biosynthetic gene cluster was discovered in *Streptomyces reseiscleroticus*. Heterologous expression of the ketosynthase, chain length factor, and acyl carrier protein in *Streptomyces lividans* confirmed that they are responsible for the assembly of a decaketide. Two regulatory genes are present in this gene cluster, including SARP-type activator SrcmRI and PadR-like repressor SrcmRII. Either overexpression of SrcmRI or disruption of SrcmRII turned on the biosynthetic pathway of chromomycins. The production titers of chromomycin A_3_/A_2_ in R5 agar in these two strains reached 8.9 ± 1.2/13.2 ± 1.6 and 49.3 ± 4.3/53.3 ± 3.6 mg/L, respectively. An engineered strain was then constructed with both SrcmRII disruption and SrcmRI overexpression, which produced chromomycins A_3_ and A_2_ in R5 agar at 69.4 ± 7.6 and 81.7 ± 7.2 mg/L, respectively. Optimization of the culture conditions further increased the titers of chromomycins A_3_ and A_2_ respectively to 145.1 ± 15.3 and 158.3 ± 15.4 mg/L in liquid fermentation.

**Conclusions:**

This work revealed the synergistic effect of manipulation of pathway repressor and activator genes in the engineering of a natural product biosynthetic pathway. The resulting engineered strain showed the highest production titers of chromomycins by a strain of *Streptomyces*, providing an efficient way to produce these pharmaceutically valuable molecules.

**Electronic supplementary material:**

The online version of this article (10.1186/s13036-018-0103-x) contains supplementary material, which is available to authorized users.

## Background

**Fig. 1:**
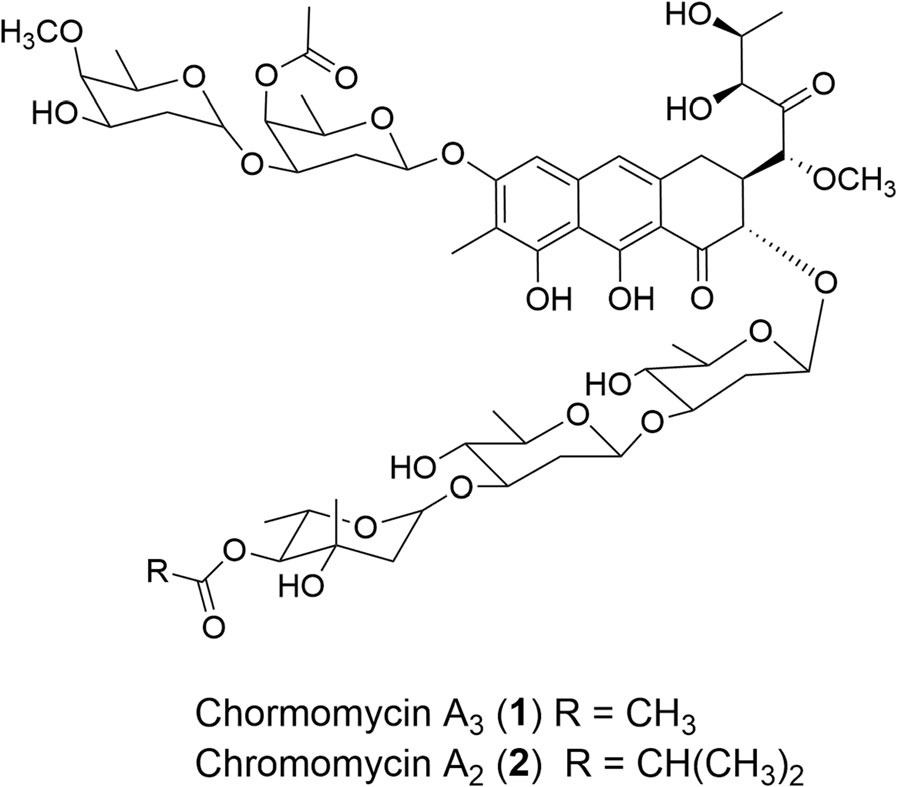
Structures of chromomycin A_3_ (**1**) and A_2_ (**2**)

Chromomycins are glycosylated polyketide compounds that were first found in *Streptomyces griseus*. These natural products belong to a group of cancerostatic and antitumor antibiotics [1]. Chromomycins and mithramycins, representative compounds of the aureolic acid family, are effective against various human tumors such as brain and testicular carcinomas [2, 3]. Chromomycin A_3_ (**1**, Fig. 1) was also reported to enable the stimulation of K562 cell erythroid differentiation [4]. It was used in the neurological therapeutics [5] and showed activities against HIV-1 [6]. **1** has been clinically used as an anticancer drug since 1960s [7]. Chromomycin A_2_ (**2**, Fig. 1) is a structurally similar analog of **1** and has shown similar bioactivities.

Antibiotic biosynthesis is typically controlled at different levels. The most fundamental level involves pleiotropic regulators which control both secondary metabolism and morphological differentiation. At the next level, regulatory genes exert their control over two or more antibiotic biosynthetic pathways in the same organism, but have no effects on morphological differentiation. Finally, the most specific level of control concerns genes encoding regulators that act only on one biosynthetic pathway [8]. These genes, whose expression is partly dependent on pleiotropic regulators, are usually found physically linked to the structural antibiotic biosynthetic genes on the chromosomes of streptomycetes. This is different from the global regulatory genes, which can be located far from the clusters they control. In most cases, the pathway-specific regulators play a positive role in the transcription of the biosynthetic genes, while others may act as transcriptional repressors. The group of proteins referred to as *Streptomyces* antibiotic regulatory proteins (SARPs) is prominent among the pathway-specific regulators in streptomycetes [8].

In Gram-positive bacteria, the phenolic acid decarboxylase (Pad) gene is involved in the phenolic acid stress response. Phenolic acids are decarboxylated by inducible Pad enzymes into vinyl phenol derivatives, which are not toxic to Gram-positive bacteria. The phenolic acid decarboxylase repressor (PadR) gene encodes the transcriptional regulator that represses genes associated with the phenolic acid stress response in Gram-positive bacteria such as *Bacillus subtilis*, *Pediococcus pentosaceus*, and *Lactobacillus plantarum* [9–11]. Among these transcriptional regulators, only a few of the PadR-like proteins have been investigated, such as AphA which activates virulence gene expression in *Vibrio cholera* [12], LmrR and LadR that inhibit the expression of a multi-drug resistance pump in *Lactococcus lactis* and *Listeria monocytogenesis*, respectively [13, 14], and CmmRII, a transcriptional repressor of chromomycin resistance/biosynthesis in *S. griseus* subsp*. griseus* [15].

*S. roseiscleroticus* (ATCC® 53903) was known to produce an antitumor drug, sultriecin [16]. The genome of this strain has been recently sequenced by our group. One putative type II polyketide synthase (PKS) gene cluster was discovered in this strain, which could be involved in the biosynthesis of chromomycins based on bioinformatics analysis. We also found two regulatory genes in the gene cluster, including the activator (*srcmRII*) and repressor (*srcmRI*) genes, which may play roles in the regulation of the whole gene cluster. By manipulating these two regulatory genes, we were able to turn on the silent chromomycin biosynthetic gene cluster, and create efficient producing strains. The production process was optimized and the titers of **1** and **2** reached 145.1 ± 15.3 and 158.3 ± 15.4 mg/L, respectively.

## Results

### Analysis of the putative chromomycin biosynthetic gene cluster

**Fig. 2:**
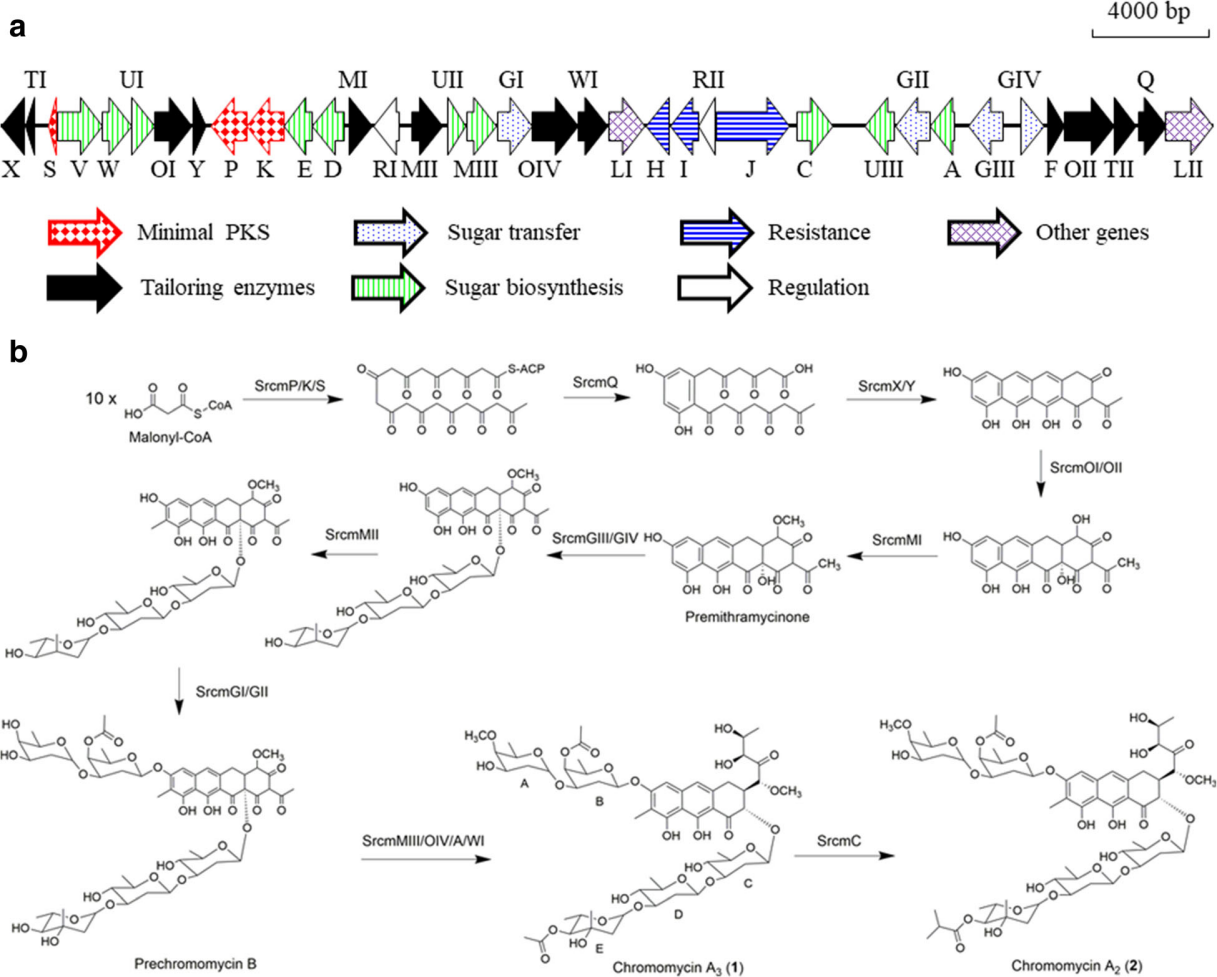
Biosynthetic gene cluster and proposed biosynthetic pathway of chromomycins. **a** Genetic organization of the *srcm* gene cluster. **b** Proposed biosynthetic pathway of chromomycins A_3_ (**1**) and A_2_ (**2**)

The genomic DNA of *Streptomyces reseiscleroticus* was sequenced by our group. Annotation of the genes revealed a type II polyketide biosynthetic gene cluster that consists of 36 genes and might be involved in the biosynthesis of **1** and **2**. Mithramycin shares the same aglycon structure with chromomycins, and its biosynthetic pathway has been well studied [17]. The putative chromomycin (*srcm*) biosynthetic gene cluster in *S. reseiscleroticus* is similar to that for mithramycin [18], and almost identical to that for chromomycin A_3_ in *S. griseus* (Fig. 2a) [1]. Three enzymes SrcmP, K and S are the minimal PKS, which might be responsible for the biosynthesis of the decaketide backbone of the chromomycin aglycon. SrcmQ, X and Y would be involved in cyclization and aromatization process. SrcmOI, OII and OIV are predicted to be oxygenases involved in the hydroxylations of aromatic polyketides. Since the chromomycin aglycon contains two methyl groups, SrcmMI and MII are proposed to be involved in these methylation steps. There are several enzymes predicted to be involved in the formation and transfer of the deoxysugar moieties, such as SrcmD, E, F, V, WI, and MIII, the glycosyltransferases SrcmGI-IV, and NDP-hexose 4-ketoreductase SrcmUI-III. The biosynthetic pathway of **1** and **2** was proposed in Fig. 2b. SrcmH, I, and J might be involved in self-resistance. SrcmRI and RII are putative regulatory proteins, likely involved in the regulation of the biosynthetic pathway of chromomycins (Table 1).

**Table 1:** Putative functions of the genes in the chromomycin biosynthetic gene cluster

Gene	Annotation	Size (aa)	Homologue	Identities
*srcmY*	cyclase	258	DacN [*Dactylosporangium sp. SC14051*]	70%
*srcmTI*	ketoreductase	258	CmmTI [*Streptomyces griseus* subsp. *griseus*]	64%
*srcmS*	acyl carrier protein	85	Acyl carrier protein [*Streptomyces* sp. 275]	100%
*srcmV*	NDP-hexose 2,3-dehydratase	462	PnxS3 [*Streptomyces* sp. TA-0256]	54%
*srcmW*	NDP-hexose 3-ketoreductase	331	PokS4 [*Streptomyces diastatochromogenes*]	53%
*srcmUII*	NDP-hexose 4-ketoreductase	252	SaqR [*Micromonospora* sp. Tu 6368]	53%
*srcmOI*	FAD-dependent monooxyngease	423	CmmOI [*Streptomyces griseus* subsp. *griseus*]	70%
*srcmX*	cyclase	142	DacG [*Dactylosporangium* sp. SC14051]	60%
*srcmK*	chain length factor	402	SaqB [*Micromonospora* sp. Tu 6368]	66%
*srcmP*	ketosynthase	422	SaqA [*Micromonospora* sp. Tu 6368]	76%
*srcmE*	dTDP-glucose 4,6-dehydratase	326	CmmE [*Streptomyces griseus* subsp. *griseus*]	75%
*srcmD*	NDP-glucose synthase	355	Lct49 [*Streptomyces rishiriensis*]	57%
*srcmMIII*	sugar O-methyltransferase	252	AlmCII [*Streptomyces* sp. A1]	61%
*srcmRI*	SARP family transcriptional regulator	292	CmmRI [*Streptomyces griseus* subsp. *griseus*]	71%
*srcmMII*	C-methyltransferase	343	mtmMII [*Streptomyces argillaceus*]	57%
*srcmUI*	NDP-hexose 4-ketoreductase	254	ChaR [*Streptomyces chattanoogensis*]	54%
*srcmMI*	O-methyltransferase	344	CtcO [*Kitasatospora aureofaciens*]	51%
*srcmGI*	glycosyltransferase	394	PyrC4 [*Streptomyces rugosporus*]	41%
*srcmOIV*	FAD-dependent monooxygenase	511	mtmOIV [*Streptomyces argillaceus*]	55%
*srcmWI*	side-chain ketoreductase	326	MtmW [S*treptomyces argillaceus*]	64%
*srcmLI*	acyl CoA ligase	409	CmmLI [*Streptomyces griseus* subsp. *griseus*]	68%
*srcmH*	ABC transporter membrane protein	251	SfrB [*Streptomyces* sp. 275]	100%
*srcmI*	ABC transporter ATP-binding protein	322	SfrA [*Streptomyces* sp. 275]	100%
*srcmRII*	PadR family transcriptional regulator	186	CmmRII [*Streptomyces griseus* subsp. *griseus*]	77%
*srcmJ*	Excinuclease ABC subunit A	825	CmrX [*Streptomyces griseus* subsp. *griseus*]	79%
*srcmC*	NDP-hexose C-methyltransferase	409	CmmC [*Streptomyces griseus* subsp. *griseus*]	78%
*srcmUIII*	4-ketoreductase	335	StaK [*Streptomyces* sp. TP-A0274]	50%
*srcmGIII*	glycosyltransferase	400	ChlC7 [*Streptomyces antibioticus*]	41%
*srcmA*	acetyltransferase	422	CmmA [*Streptomyces griseus* subsp. *griseus*]	74%
*srcmGIV*	glycosyltransferase	391	PyrC4 [*Streptomyces rugosporus*]	41%
*srcmGII*	glycosyltransferase	391	SsfS6 [*Streptomyces* sp. SF2575]	38%
*srcmF*	dTDP-4-keto-6-deoxy-D-glucose epimerase	199	dTDP-4-keto-6-deoxy-D-glucose epimerase [*Streptomyces tsukubensis*]	78%
*srcmOII*	oxygenase	550	DacO2 [*Dactylosporangium* sp. SC14051]	57%
*srcmTII*	ketoreductase	253	RubJ [*Streptomyces collinus*]	50%
*srcmQ*	aromatase	320	DacK [*Dactylosporangium* sp. SC14051]	45%
*srcmLII*	acyl CoA ligase	536	SsfL2 [*Streptomyces* sp. SF2575]	48%

### Heterologous expression of the type II minimal PKS in *S. lividans* K4–114

**Fig. 3:**
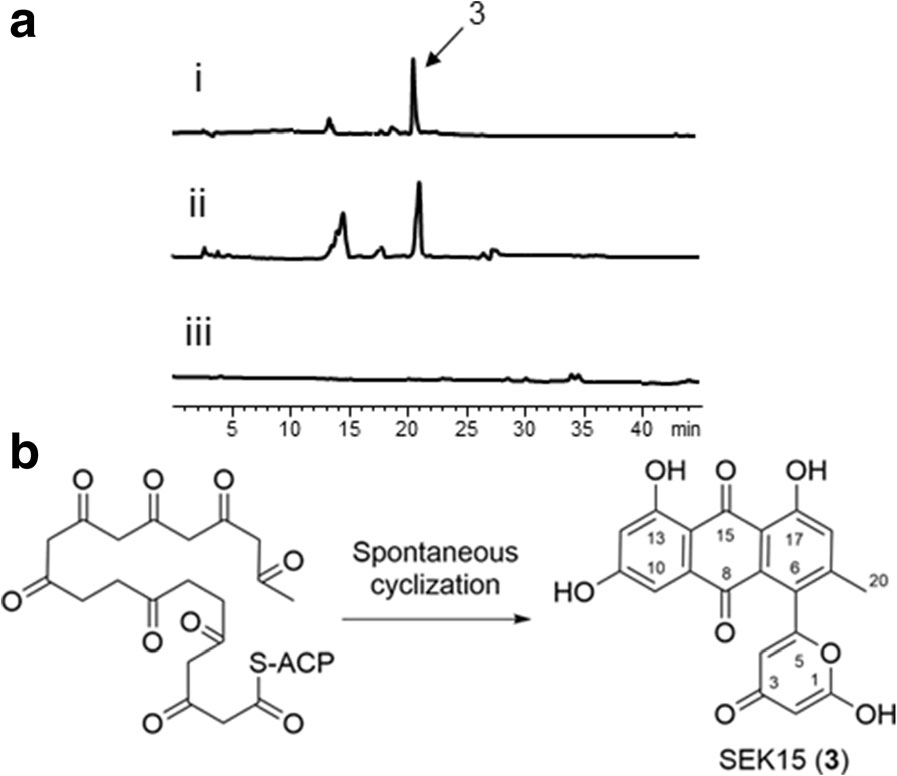
Heterologous expression of SrcmPKS in *S. lividans* K4–114. **a** HPLC analysis of the extracts of *S. lividans* K4–114 harboring pSUN6 (i), pTZ3 (ii) and empty pRM5 vector (iii) respectively. Compounds were detected at 420 nm. **b** Formation of SEK15b (**3**) through spontaneous cyclization of the nascent decaketide chain

According to the annotation of the gene cluster in *S. roseiscleroticus*, *srcmPKS* are putative minimal PKS genes in chromomycin biosynthesis. In order to confirm the function of SrcmPKS, we constructed a plasmid pRM5-*srcmPKS* (pSUN6) and introduced it into *S. lividans* K4–114 for heterologous expression. As shown in trace i of Fig. 3a, a major product **3** at 21.6 min was observed in the extract of engineered strain KS1. For the reference, we constructed another pRM5-based expression plasmid (pTZ3) that carries the *OxyABC* genes encoding the oxytetracycline minimal PKS in *Streptomyces rimosus*. As shown in trace ii of Fig. 3a, a peak was eluted at the same time as **3**, whereas *S. lividans* K4–114 harboring the empty vector pRM5 did not produce any polyketide products (trace iii, Fig. 3a). The oxytetracycline minimal PKS OxyABC was previously reported to synthesize a decaketide which will be spontaneously cyclized into SEK15b (Fig. 3b) [19]. These results indicated that SrcmPKS has the same function as OxyABC.

In order to isolate and identify compound **3**, a scaled up culture of *S. lividans* K4–114/pSUN6 was prepared. ESI-MS analysis of **3** showed ion peaks [M + H]^+^ at *m*/*z* 381.0 and [M - H]^−^ *m*/*z* 379.1 (Additional file 1: Figure S1a), indicating that the molecular weight of this compound is 380, which corresponds to that of SEK15b. The ^1^H NMR (Additional file 1: Table S1) of **3** indicated that the compound has five aromatic proton signals and one high field methyl group signal. Twenty carbons were observed in the ^13^C NMR spectrum (Additional file 1: Table S1) of **3**. After a comparison with the published data [20], compound **3** was identified as SEK15b. Therefore, SrcmPKS were confirmed to be the minimal PKS for the biosynthesis of a decaketide.

### Disruption of *srcmRII* in *S. roseiscleroticus* and products characterization

**Fig. 4:**
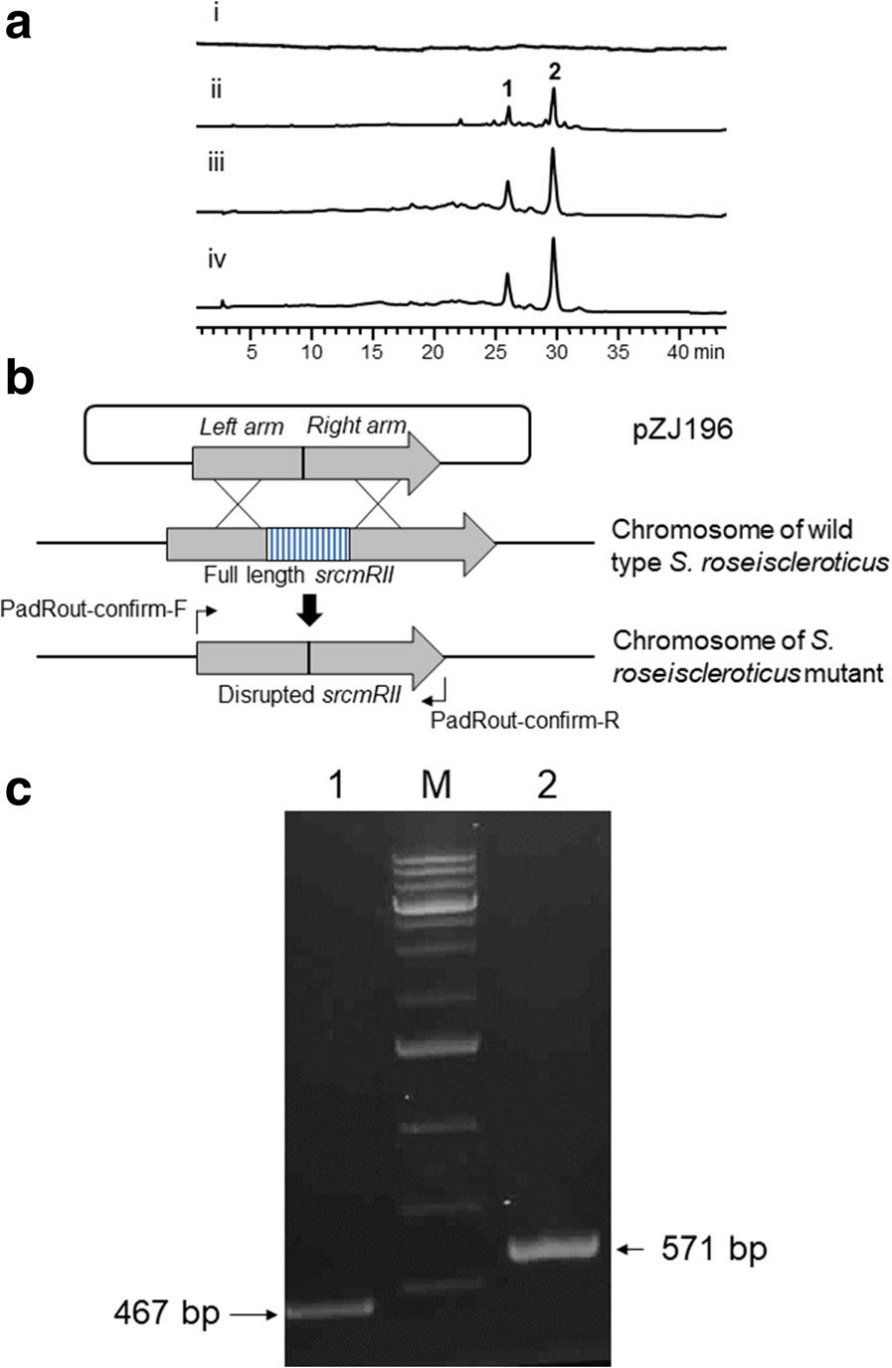
Engineered production of chromomycins in *S. roseiscleroticus*. **a** HPLC analysis (420 nm) of chromomycins production by wild type *S. roseiscleroticus* (i), *S. roseiscleroticus* SR2 (ii, *srcmRII* disruption), *S. roseiscleroticus* SR1 (iii, *srcmRI* overexpression), *S. roseiscleroticus* SR3 (iv, *srcmRI* overexpression and *srcmRII* disruption) in R5 agar. **b** The disruption strategy of *scrmRII* in *S. roseiscleroticus* using a double crossover homologous recombination approach. **c** PCR conformation of the double crossover mutant (*S. roseiscleroticus*/Δ*scrmRII*). M: 1 kb plus DNA ladder; 1: PCR product from the mutant; 2: PCR product from the wild type

After we confirmed that the decaketide was synthesized by SrcmPKS, we sought to search for chromomycins in the extract of wild type *S. roseiscleroticus*. However, there were no target peaks observed (trace i, Fig. 4a). Thus, this gene cluster was considered silent in *S. roseiscleroticus* under the lab conditions.

Regulatory genes play important roles in controlling the expression of antibiotic biosynthetic pathways. *srcmRII* is one of the regulatory genes in the chromomycin biosynthetic gene cluster. BLAST analysis indicated that this gene encodes a PadR-like family transcriptional regulator, and is a putative repressor. To identify the role of *srcmRII* in chromomycin biosynthesis, we attempted to inactivate *srcmRII* in *S. roseiscleroticus* using a double crossover homologous recombination approach (Fig. 4b). To this end, we constructed pZJ196 that is a derivative of pKC1139 and harbors the homologous arms flanking *srcmRII*. After the transformation of *S. roseiscleroticus* with this plasmid through conjugation and subsequent homologous recombination, an engineered strain SR2 was obtained. PCR analysis showed that a short version of *srcmRII* was amplified from the mutant (Fig. 4c), confirming that this gene was successfully disrupted. This engineered strain was cultured onto R5 agar medium, with wild type *S. roseiscleroticus* as control. HPLC and ESI-MS analyses revealed that the wild type strain did not produce chromomycins in R5 agar, while the engineered strain SR2 produced two new main products (traces i and ii, Fig. 4a).

The two main products **1** and **2** were purified and obtained as yellow powder. They were identified as chromomycins A_3_ and A_2_, respectively, based on spectroscopic analyses. Their molecular weights were determined to be 1210 ([M-H]^−^ *m/z* 1209.3, [M + Na]^+^ *m/z* 1233.4) and 1182 ([M-H]^−^ *m/z* 1181.3, [M + Na]^+^ *m/z* 1205.4), respectively, based on the ESI-MS spectra (Additional file 1: Figure S1b-c). Their UV absorptions (Additional file 1: Figure S1b-c) were same as the previously reported [4]. The structures were further confirmed by comparing the ^1^H NMR data (Additional file 1: Table S2) with those in the literature [21, 22]. In order to calculate the production of **1** and **2**, we established a standard curve for the relationship between the amounts of each pure compound (μg) and the peak areas (420 nm). Based on the standard curve, the titers of **1** and **2** in strain SR2 in R5 agar were determined to be 49.3 ± 4.3 and 53.3 ± 3.6 mg/L, respectively.

### Overexpression of SrcmRI in *S. roseiscleroticus*

Besides *srcmRII*, the other regulatory gene *srcmRI* is a putative SARP, which was predicted to be the activator of the chromomycin biosynthetic pathway. To find out the role of *srcmRI* in the biosynthesis of chromomycins, we attempted to overexpress the *srcmRI* gene in *S. roseiscleroticus*. To this end, we constructed a pSET152-derived plasmid pZJ183 that harbors *srcmRI* under the strong constitutive promoter *ermP*. After the transformation of *S. roseiscleroticus* with pZJ183, we obtained the engineered strain SR1. The culture of this strain in R5 agar was extracted and analyzed as described before. As shown in trace iii of Fig. 4a, compared to the wild type control (trace i, Fig. 4a), strain SR1 in R5 agar also produced **1** and **2**. The average titers of **1** and **2** were 8.9 ± 1.2 and 13.2 ± 1.6 mg/L, respectively.

### Optimization of the production of **1** and **2** in engineered *S. roseiscleroticus*

According to our results, either inactivation of *srcmRII* or overexpression of the *srcmRI* gene in *S. roseiscleroticus* “turned on” the chromomycin biosynthetic pathway, and led to the production of **1** and **2** at different titers. In order to further improve the production of **1** and **2**, we tried overexpressing *srcmRI* in strain SR2, in which *srcmRII* was disrupted. The double crossover strain SR2 was used as host, and transformed with pZJ183 to get strain SR3, which has both disrupted *srcmRII* and overexpressed *srcmRI*.

We cultured the engineered strain SR3 in R5 agar supplemented with apramycin, as described above, and the culture was extracted and analyzed. As shown in trace iv of Fig. 4a, strain SR3 also produced **1** and **2**. The titers of **1** and **2** were 69.4 ± 7.6 and 81.7 ± 7.2 mg/L, respectively. Compared to SR2 strain under the same culture conditions, the SR3 strain produced approximately 48% more chromomycins (**1** and **2**). Strain SR3 produced approximately 7.5 folds more than SR1 did.

**Fig. 5:**
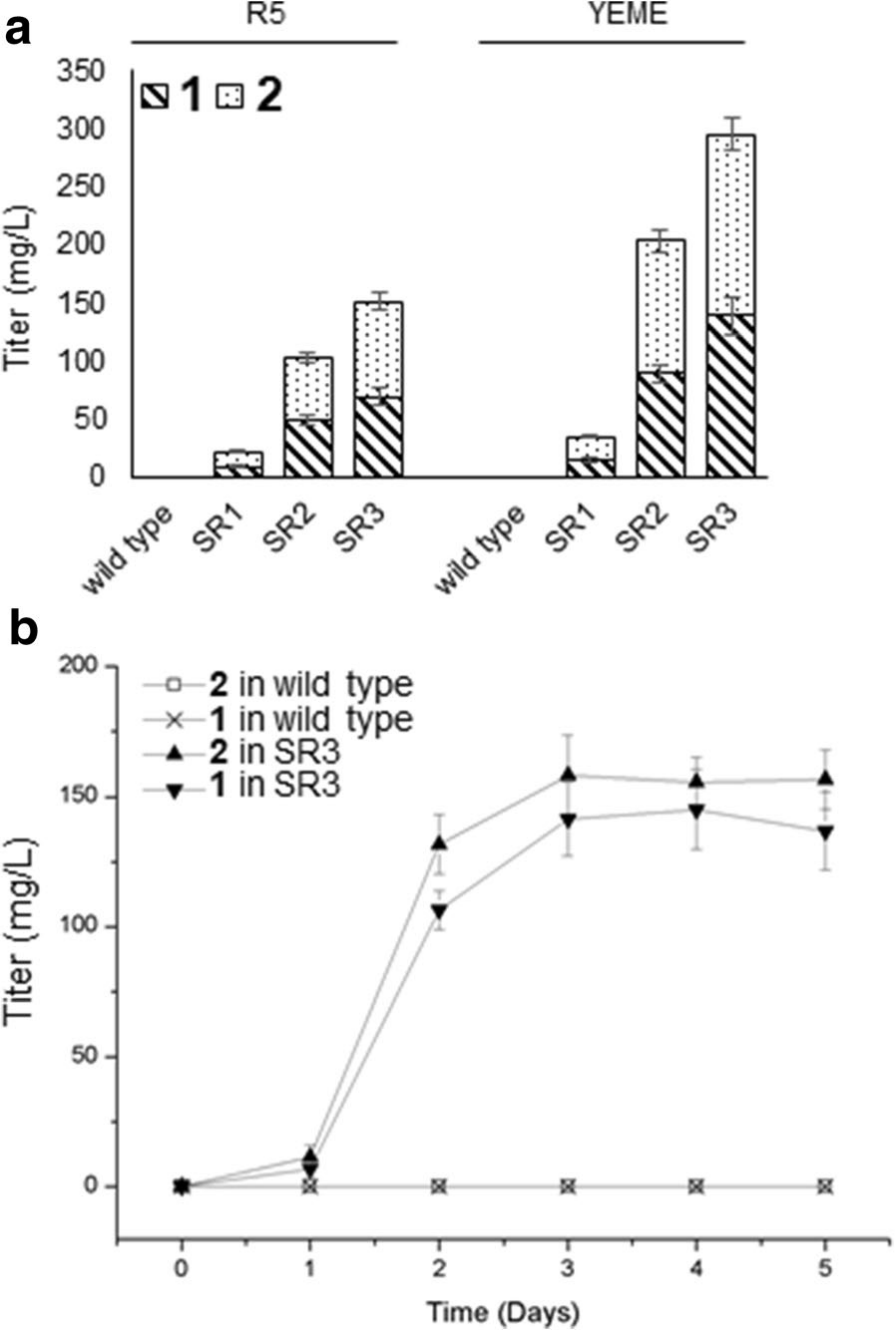
Optimization of chromomycins production. **a** Production of **1** and **2** in two different solid media. **b** Time course analysis of chromomycins production of in liquid YEME medium. Error bars represent the standard deviation from three replicates

Since malonyl-CoA is the substrate for assembly of the polyketide aglycon, more sugar in the medium might provide more carbon substrate for chromomycin biosynthesis. Additionally, sugar moieties are also present in the side chains of chromomycins. In order to provide more sugar in the medium, we attempted to use the yeast extract-malt extract (YEME) medium (containing 1% glucose and 17% sucrose) for chromomycins production. Three engineered strains SR1, SR2 and SR3 were cultured on YEME solid medium for 7 days. As shown in Fig. 5a, the production of **1** and **2** was significantly increased in the YEME culture. Compared to the cultures in R5 agar, the production (**1** plus **2**) in YEME was much higher, with a 58.3% increase for SR1, 98.9% increase for SR2, and 95.8% increase for SR3. The titers of **1** and **2** in SR3 were 138.5 ± 15.6 and 157.3 ± 13.8 mg/L, respectively.

These results further confirmed that SR3, with *srcmRI* overexpression and *srcmRII* disruption, has higher production of chromomycins than the other two strains. Considering further application of these engineered strains in industry, we cultured SR3 in YEME liquid medium for time course analysis. The HPLC results (Fig. 5b) showed that the titer has been increasing in the first 3 days, and then reached a stable level. The highest titer of **1** was 145.1 ± 15.3 mg/L, and that of **2** was 158.3 ± 15.4 mg/L. This engineered strain provides a great starting strain for efficient production of these two products in the pharmaceutical industry.

## Discussion

The genomic DNA of *S. reseiscleroticus* was sequenced by our group, and a gene cluster of 36 genes were predicted to be involved in the biosynthesis of chromomycins. The proposed biosynthetic pathway of **1** and **2** is shown in Fig. 2b. The minimal PKS consisting of SrcmP (ketosynthase), SrcmK (polyketide chain length factor) and SrcmS (acyl carrier protein) was proposed to synthesize a decaketide backbone. Heterologous expression of SrcmP, K and S in *S. lividans* yielded **3**, a spontaneously cyclized decaketide product, confirming that the *srcm* minimal PKS synthesizes a decaketide chain for chromomycin biosynthesis. The nascent decaketide backbone will be cyclized with the participation of the aromatase SrcmQ to form the first ring, which will be further folded and modified by the cyclases (SrcmX and SrcmY) and ketoreductases (SrcmTI and SrcmTII) to form a four-ring structure. Two hydroxyl groups will be introduced by the oxygenases SrcmOI and SrcmOII to form 4-demethyl-premithramycinone. The methylation on 1′-hydroxy group is proposed to be catalyzed by SrcmMI, a putative O-methyltransferase, leading to the synthesis of premithramycinone. After the synthesis of aglycon part, several steps of glycosylation will occur. Additional structural modifications by other tailoring enzymes and the four glycosyltransferases (SrcmGI-IV) afford the ultimate structure of **1** and **2** (Fig. 2b). The type II polyketide biosynthetic gene cluster we found in *S. reseiscleroticus* is fully consistent with the previously reported chromomycin biosynthetic gene cluster [1]. While we confirmed the functions of the minimal PKS in chromomycin biosynthesis in the present work, the remaining biosynthetic enzymes can be further characterized through heterologous expression or gene knockout in the future work. Understanding of these enzymes and corresponding biosynthetic steps may further facilitate rational engineering of the chromomycin biosynthetic pathway for enhanced production of these pharmaceutically important molecules or generation of new chromomycin analogs.

The *srcm* gene cluster contains two putative regulatory genes, respectively encoding a SARP-type activator SrcmRI and a PadR-like repressor SrcmRII which was predicted to be a transcriptional inhibitor in chromomycin resistance/biosynthesis in the producing microorganism [1, 15]. It was previously reported that disruption of PadR-like repressor CmmRII increased the production of **1** by approximately 70% [15]. Overexpression of the SARP-type activator gene *asm18* in *Actinosynnema pretiosum* increased the titer of N-demethyl-4, 5-desepoxy-maytansinol to 50 mg/L, which was increased by 4.7-fold compared to the wild type strain [23]. Similarly, *mtmR* from *Streptomyces argillaceus* was identified as a SARP regulatory gene. Replacement of this gene with an antibiotic resistance gene abolished the production of mithramycin, while overexpression of *mtmR* in *S. argillaceus* led to a 16-fold increase in mithramycin production [18]. In *S. roseiscleroticus*, we identified two regulatory genes in the *scrm* biosynthetic gene cluster, and tried overexpression of SrcmRI and deletion of SrcmRII to enable and increase the production of chromomycins. Overexpression of SrcmRI turned the wild type to an efficient chromomycin producer (22.1 mg/L of chromomycins). Disruption of the repressor SrcmRII showed an even more significant effect on chromomycins production (102.6 mg/L of chromomycins). This phenomenon suggests that SrcmRII has high activity on the inhibition of the biosynthetic pathway, which also explains why the wild type does not produce chromomycins under lab conditions. The silent chromomycin biosynthetic pathway was activated by disrupting the PadR-like repressor SrcmRII or overexpressing the SARP-type activator SrcmRI in *S. roseiscleroticus*. It was also found that combination of SrcmRII disruption and SrcmRII overexpression showed a synergistic effect that further increased the production titers and yielded a more efficient producing strain of chromomycins. This work thus demonstrates a new method to improve the production of target products by simultaneously manipulating both the pathway activator and repressor.

## Conclusions

In conclusion, we sequenced the genome of *S. reseiscleroticus* and discovered a type II polyketide biosynthetic gene cluster consisting of 36 open reading frames, which was predicted to be involved in chromomycin biosynthesis. Heterologous expression of the minimal PKS encoded by *srcmPKS* in *S. lividans* K4–114 confirmed that these enzymes work collaboratively to synthesize the decaketide backbone of chromomycins. In the *srcm* gene cluster, two putative regulatory genes were identified, including *srcmRI* encoding a SARP-type activator and *srcmRII* encoding a PadR-like repressor. Overexpression of SrcmRI activated the chromomycin biosynthetic pathway and biosynthesized **1** and **2**, whereas the disruption of SrcmRII increased the production much more than SrcmRI overexpression. In order to optimize the production of chromomycins, another engineered strain SR3 was constructed by both overexpressing SrcmRI and disrupting SrcmRII. Under the optimized culture conditions, **1** was produced at 145.1 ± 15.3 mg/L and **2** at 158.3 ± 15.4 mg/L. Therefore, the inactivation of PadR-like repressor SrcmRII and overexpression of the SARP-type activator SrcmRI transformed *S. roseiscleroticus* from a non-producing strain into an efficient producer of **1** and **2**. The engineered strain showed the promise of application in the pharmaceutical industry.

## Methods

### General method

Products were analyzed and purified on an Agilent 1200 HPLC instrument. ESI-MS spectra were obtained on an Agilent 6130 quadrupole LC-MS. NMR spectra were recorded on a JEOL NMR instrument (300 MHz for ^1^H NMR, 75 MHz for ^13^C NMR). NMR spectra were collected in CDCl_3_ for **1** and **2**, and in DMSO-*d*_*6*_ for **3.** The chemical shift (*δ*) values are given in parts per million (ppm). The coupling constants (*J* values) are reported in Hertz (Hz).

### Strains, culture conditions, and plasmids

*E. coli* DH5α and ET12567 were grown in Luria-Bertani broth. *S. roseiscleroticus* (ATCC^®^ 53903) and *S. lividans* K4–114 [24] was cultured in ISP4 medium for seed culture preparation. R5 agar and YEME medium were used as the fermentation medium for chromomycins production, and TSBY salt medium for double crossover [10, 16]. For conjugation, the *Streptomyces* strains were grown in TSB medium. R5 medium was used for genomic DNA isolation. Plasmid pRM5 [25] was used for heterologous gene expression in *S. lividans* K4–114. For gene manipulations in *S. roseiscleroticus*, plasmid pSET152 [26] was used for gene overexpression, and pKC1139 [26] for gene disruption. The concentrations of antibiotics used were 50 μg/mL for ampicillin, 50 μg/mL for apramycin, 50 μg/mL for thiostrepton, and 50 μg/mL for nalidixic acid.

### DNA manipulation and sequence analysis

PCRs were performed using the Phusion DNA polymerase (Thermo Scientific, USA). *S. roseiscleroticus* and *Streptomyces rimosus* genomes were isolated by a standard procedure [27]. Conjugation was performed as described previously [28]. Homologous sequence database search and multiple sequence alignments were executed with BLASTP and ClustalX. The gene cluster was annotated with FramePlot [29] and was deposited in GenBank under accession number MG975976.

### Heterologous expression of minimal PKS in *S. lividans* K4–114

The minimal PKS genes *srcmPKS* were cloned from *S. roseiscleroticus* genome. The *srcmPK* genes were amplified using PCR with the primers KS-F-PacI and CLF-R-EcoRI-NheI (Table 2) and ligated into the pJET1.2 cloning vector (Thermo Fisher Scientific) to yield pZJ180 (Table 3). We amplified the *srcmS* gene with primers ACP-F-SpeI and ACP-R-NheI (Table 3) and ligated it into pJET1.2 to yield pZJ172 (Table 3). After sequencing, *srcmS* was excised from pZJ172 with SpeI and NheI and ligated into pZJ180 at the *Nhe*I site to yield pZJ178 (Table 3). The entire minimal PKS gene cassette was then cut from pZJ178 using *Pac*I and *Nhe*I and ligated into pRM5 (with a thiostrepton resistance gene) to yield pSUN6 (Table 3). Introduction of pSUN6 into the host strain *S. lividans* K4–114 via protoplast transformation afforded strain KS1 (Table 3). Using the same cloning strategy, the *oxyAB* genes were amplified from the genomic DNA of *S. rimosus* with primers OxyA-5-PacI and OxyB-3-NheI, and the *oxyC* gene was amplified using PCR with primers OxyC-5-SpeI and OxyC-3-NheI (Table 2). These genes were combined in pJET1.2 and ligated into pRM5 between *Pac*I and *Nhe*I to yield pTZ3 (Table 3).

**Table 2:** List of primers used in this study^a^

Primers	Sequence
KS-F-PacI	5’-AATTAATTAAGGAGGAGCCCATATGAGAAGGCGCGTCGTGGT-3’
CLF-R-EcoRI-NheI	5’-AAGAATTCGCTAGCCTACGGTCCCGACAGCGCCA-3’
OxyA-5-PacI	5’-AATTAATTAAGGAGGAGCCCATATGTCCAAGATCCATGACGC-3’
OxyB-3-NheI	5’-AAGCTAGCTCAGTCCCGGCCGCTGACCA-3’
OxyC-5-SpeI	5’-AAACTAGTGGAGGAGCCCATATGACCCTGCTCACCCTCTC-3’
OxyC-3-NheI	5’-AAGCTAGCTCACTTGTCCCGCGCGGCGC-3’
ACP-F-SpeI	5’-AAACTAGTGGAGGAGCCCATATGATGACGGTGGACGATCT-3’
ACP-R-NheI	5′- AAGCTAGCTCAGCCCACCCCGATGCCGT-3’
14–2-SARP-F-XbaI-NdeI	5′- AATCTAGAGGAGGAGCCCATATGAGCTCCGACAGCGACTGCA-3’
14–2-SARP-R-XbaI	5′- AATCTAGATCAGCCGGCCCGGCGCTGCC-3’
PadRout-left-F-EcoRI	5′- AAA GAATTC TACTCGCCGGAGTGCGCACGGTCGAA-3’
PadRout-left-R-XbaI	5′- AAA TCTAGA CTTCTTCGCGCAGCTTGCGCAGCT-3’
PadRout-right-F-XbaI	5′- AAA TCTAGA CGTACCGCTGTCGCCGCGAT-3’
PadRout-right-R-HindIII	5′- AAA AAGCTT ATGATCTCGATCATCGAGCG-3’
PadRout-confirm-F	5’-AACATATGGCTCTGGGCACGCTGCA-3’
PadRout-confirm-R	5’-AAGAATTCTCACTGCGGGTCCTCCTGCG-3’

^a^The restriction sites are underlined

**Table 3:** List of strains and plasmids used in this work

Strains	Genotype	Source
*S. roseiscleroticus*	Wild type	ATCC
*S. lividans* K4–114	*pro-2 str-6SLP2 − SLP3 − act::ermE Streptomyces*	[24]
SR1	*S. roseiscleroticus*/pZJ183 (*srcmRI* overexpression)	This Work
SR2	*S. roseiscleroticus*/pZJ196 (*srcmRII* disruption)	This Work
SR3	*S. roseiscleroticus*/pZJ183 + pZJ196 (*srcmRI* overexpression and *srcmRII* disruption)	This Work
KS1	*S. lividans* K4–114/pSUN6	This Work
Plasmids	Description	Source
pZJ172	*srcmS in pJET1.2*	This Work
pZJ178	*srcmPKS in pJET1.2*	This Work
pZJ179	*srcmRI in pJET1.2*	This Work
pZJ180	*rcmPK in pJET1.2*	This Work
pZJ178	*srcmPKS in pJET1.2*	This Work
pZJ179	*srcmRI in pJET1.2*	This Work
pSUN6	*srcmPKS in pRM5*	This Work
pTZ3	*oxyABC in pRM5*	This Work
pZJ183	*srcmRI in pSET152*	This Work
pZJ191	*srcmRII knockout left arm in pJET1.2*	This Work
pZJ192	*srcmRII knockout right arm in pJET1.2*	This Work
pZJ195	*srcmRII knockout left arm in pKC1139*	This Work
pZJ196	*srcmRII knockout left arm-right arm in pKC1139*	This Work

### Overexpression of srcmRI in *S. roseiscleroticus*

This *srcmRI* gene was PCR amplified with primers 14–2-SARP-F-XbaI-NdeI and 14–2-SARP-R-XbaI (Table 2) and ligated into pJET1.2 to yield pZJ179 (Table 3). After sequencing, the gene was excised from pZJ179 and ligated into pSET152 (with an apramycin resistance gene) at the *Xba*I site under the *ermE* promoter to yield pZJ183. The *Nde*I site included in the forward primer was used to determine the correct direction. Introduction of pZJ183 (Table 3) into wild type *S. roseiscleroticus* by protoplast transformation afforded the recombinant strain SR1 (Table 3), in which the expression of *srcmRI* is under the control of the constitutive *ermE* promoter [27].

### Gene disruption of srcmRII in *S. roseiscleroticus*

The *srcmRII* gene was inactivated by gene replacement to construct a ∆*srcmRII* mutant. The homologous arms were amplified with two sets of primers. PadRout-left-F-EcoRI and PadRout-left-R-XbaI (Table 2) were used to amplify the left arm, which was ligated into pJET1.2 to yield pZJ191 (Table 3). The fragment was subsequently ligated into pKC1139 at *EcoR*I/*Xba*I sites to yield pZJ195 (Table 3). PadRout-right-F-XbaI and PadRout-right-R-HindIII (Table 2) were used to amplify the right arm, which was cloned into pJET1.2 to yield pZJ192 (Table 3). The right arm was excised from pZJ192 with *Xba*I and *Hind*III and subsequently ligated into pZJ195 between the same sites to yield pZJ196 (Table 3). After introduction of pZJ196 into wild type *S. roseiscleroticus* strain via *E. coli*-*S. roseiscleroticus* conjugation, transformants were cultured at 37 °C to screen for an apramycin-resistant phenotype to identify single crossover mutants. The single crossover mutants were cultured on blank TSBY salt plates for several generations, and then transferred onto the TSBY salt plates with or without apramycin. After 7 days’ culture, the TSBY plates were screened for the double crossover colonies, which should have lost the apramycin-resistant phenotype. The strains that grew on blank TSBY salt plates, but did not grow with apramycin, were selected as double crossover candidates. The double crossover candidates were further confirmed by PCR with primers PadRout-confirm-F and PadRout-confirm-R (Table 2). The PCR product was also sequenced for further confirmation. After the double crossover strain was confirmed, it was named as SR2. pZJ183 was transferred into the SR2 strain via conjugation to yield SR3, which had both *srcmRII* disruption and *srcmRI* overexpression.

### Detection and purification of chromomycin A_3_ (**1**), chromomycin A_2_ (**2**) and SEK15b (**3**)

The engineered *S. roseiscleroticus* or *S. lividans* K4–114 strains grown on solid media were extracted with the method described before [1]. The liquid culture broths of the *S. roseiscleroticus* or *S. lividans* K4–114 strains were first centrifuged at 3000×g for 10 min to harvest the cells. The supernatants were extracted three times with an equal volume of ethyl acetate, and the pellet cells were extracted with methanol. The two extracts were combined and dried with rotavapor. The residues were dissolved in 10 mL of methanol for HPLC analysis. The products were analyzed on an Agilent 1200 HPLC instrument (Agilent eclipse plus C_18_, 5 μm, 4.6 mm × 250 mm) using a linear gradient of 10 to 90% acetonitrile-water (containing 0.1% trifluoroacetic acid) over 45 min at a flow rate of 1 mL/min.

To establish the standard curves, chromomycins were purified from 500 mL of culture of strain SR2 in R5 agar. The crude extract was fractionated using a silica gel-60 column, eluted with different ratios of methanol-chloroform (0:100, 1:99, 2:98, 3:97, 5:95, 10:90, 15:85, *v*/v, each 250 mL) to afford seven fractions. The fractions containing the target compounds were further separated by HPLC (Agilent Eclipse Plus C_18_, 5 μm, 4.6 mm × 250 mm), eluted with acetonitrile-water at a flow rate of 1 mL min^− 1^. **2**, found in fraction 2, was further purified using HPLC with an isocratic elution of 47% acetonitrile-water. The target peak at 15.4 min was collected to yield 22.3 mg of **2** in pure form. **1** was found in fraction 3 and was purified by HPLC with an isocratic elution of 43% acetonitrile-water. The target peak at 13.6 min was collected to yield 10.5 mg of **1** in pure form.

The pure compounds were dissolved in 1 mL of methanol:DMSO (*v*/v 50:50), which were serially diluted to obtain different concentrations. For each concentration, 100 μL of these samples were injected into the HPLC to obtain the peak areas to establish standard curves for **1** and **2**. Standard curves based on the linear relationship between the amounts (μg) and the peak areas (420 nm) were established for **1** (y = 114.38× - 853.13, R^2^ = 0.9965) and **2** (y = 44.436× + 196.1, R^2^ = 0.9912) for quantification of their production titers in different engineered strains. **3** was extracted from 500 mL of liquid culture broth of strain KS1 with an equal volume of ethyl acetate for three times. After evaporation of the solvent, the crude extract was also fractionated on a silica gel-60 column using the same procedure as described above. **3** was observed in fraction 4. It was purified using HPLC by an isocratic elution with 37% acetonitrile-water. The target peak at 17.1 min was collected to yield 9.1 mg of **3** in pure form.

### Chromomycins production in liquid and solid media

For the time-course analysis, 10^6^/mL spores of wild type *S. roseiscleroticus* or the engineered strain SR3 were inoculated respectively into 500 mL of liquid YEME with apramycin in a 2-L flask at 28 °C. The fermentation broths were sampled (10 mL) at 0, 1, 2, 3, 4 and 5 days and the products were analyzed by HPLC using the above-mentioned method. For production analysis in solid medium, 10^6^/mL spores of wild type *S. roseiscleroticus*, engineered strains SR1, SR2 and SR3 were inoculated respectively onto R5 agar or YEME solid medium, and incubated at 28 °C for 7 days.

## Additional file

Additional file 1: **Figure S1.** UV and ESI-MS spectra of SEK15b (**3**, a), chromomycin A_3_ (**1**, b) and chromomycin A_2_ (**2**, c). **Table S1.** The ^1^H (300 MHz) and ^13^C NMR (75 MHz) data for SEK15b (**3**) (DMSO-*d*_*6*_, *δ* in ppm, *J* in Hz). **Table S2.** The ^1^H NMR (300 MHz) data for chromomycins A_3_ (**1**) and A_2_ (**2**) (CDCl_3_, *δ* in ppm, *J* in Hz). (DOCX 200 kb)

## Abbreviations

Pad: Phenolic acid decarboxylase
PKS: Polyketide synthase
SARP: *Streptomyces* antibiotic regulatory protein
YEME: Yeast extract-malt extract
