# Supplementary material for: Manipulation of two regulatory genes for efficient production of chromomycins in Streptomyces reseiscleroticus
Source: J Biol Eng. 2018 Jun 7;12:9. doi: 10.1186/s13036-018-0103-x (PMC5992853; doi:10.1186/s13036-018-0103-x)
Supplement: Supplementary file 1 — Figure S1. UV and ESI-MS spectra of SEK15b (3, a), chromomycin A3 (1, b) and chromomycin A2 (2, c). Table S1. The 1H (300 MHz) and 13C NMR (75 MHz) data for SEK15b (3) (DMSO-d6, δ in ppm, J in Hz). Table S2. The 1H NMR (300 MHz) data for chromomycins A3 (1) and A2 (2) (CDCl3, δ in ppm, J in Hz). (DOCX 200 kb) [file 13036_2018_103_MOESM1_ESM.docx]

**Supporting Information**

**for**

**Manipulation of two regulatory genes for efficient production of chromomycins in *Streptomyces reseiscleroticus***

Lei Sun^1^, Jia Zeng^1^, Peiwu Cui^2^, Wei Wang^2^, Dayu Yu^3^ and Jixun Zhan^1,2*^

1 Department of Biological Engineering, Utah State University, 4105 Old Main Hill, Logan, UT 84322-4105, USA

2 TCM and Ethnomedicine Innovation & Development Laboratory, School of Pharmacy, Hunan University of Chinese Medicine, Changsha, Hunan 410208, China

3 Hangzhou Viablife Biotech Co., Ltd., 1 Jingyi Road, Yuhang District, Hangzhou, Zhejiang 311113, China

**Correspondence:** [jixun.zhan@usu.edu](mailto:jixun.zhan@usu.edu).

| **a** | **** | **** | **** |
| --- | --- | --- | --- |
| **b** | **** | **** | **** |
| **c** | **** | **** | **** |

**Figure S1**. UV and ESI-MS spectra of SEK15b (**3**, a), chromomycin A_3_ (**1**, b) and chromomycin A_2_ (**2**, c).

**Table S1.** The ^1^H (300 MHz) and ^13^C NMR (75 MHz) data for SEK15b (**3**) (DMSO-*d_6_*, *δ* in ppm, *J* in Hz).

| Position | ^13^C | ^1^H |
| --- | --- | --- |
| 1 | 164.5 |  |
| 1-OH |  | 11.98 (1H, s) |
| 2 | 89.7 | 5.46 (1H, s) |
| 3 | 170.1 |  |
| 4 | 105.5 | 6.23 (1H, s) |
| 5 | 157.8 |  |
| 6 | 127.4 |  |
| 7 | 136.9 |  |
| 8 | 181.9 |  |
| 9 | 134.1 |  |
| 10 | 107.2 | 7.07 (1H, s) |
| 11 | 164.5 |  |
| 11-OH |  | 11.55 (1H, s) |
| 12 | 108.4 | 6.63 (1H, s) |
| 13 | 164.2 |  |
| 13-OH |  | 13.22 (1H, s) |
| 14 | 110.3 |  |
| 15 | 187.9 |  |
| 16 | 122.5 |  |
| 17 | 160.2 |  |
| 17-OH |  | 11.11 (1H, s) |
| 18 | 112.2 | 7.67 (1H, s) |
| 19 | 143.8 |  |
| 20 | 20.0 | 2.69 (3H, s) |

**Table S2.** The ^1^H NMR (300 MHz) data for chromomycins A_3_ (**1**) and A_2_ (**2**) (CDCl_3_, *δ* in ppm, *J* in Hz).

| Position | **3** | | **4** | |
| --- | --- | --- | --- | --- |
| Aglycon |  | |  |  |
| 2-H | 4.73 (1H, brs) | | 4.73 (1H, brs) | |
| 3-H | 2.60-2.65 (1H, m) | | 2.60-2.65 (1H, m) | |
| 4-H_ax_ | 3.09-3.13 (1H, m) | | 3.08-3.12 (1H, m) | |
| 4-H_eq_ | 2.65-2.67 (1H, m) | | 2.67-2.71 (1H, m) | |
| 5-H | 6.66 (1H, s) | | 6.66 (1H, s) | |
| 7-CH_3_ | 2.19 (3H, s) | | 2.18 (3H, s) | |
| 8-OH | 9.83 (1H, s) | | 9.83 (1H, s) | |
| 9-OH | 15.72 (1H, s) | | 15.72 (1H, s) | |
| 10-H | 6.77 (1H, s) | | 6.78 (1H, s) | |
| 1'-H | 4.73 (1H, brs) | | 4.73 (1H, brs) | |
| 1'-OCH_3_ | 3.53 (3H, s) | | 3.53 (3H, s) | |
| 3'-H | 4.23 (1H, brs) | | 4.24 (1H, brs) | |
| 3'-OH | 3.69-3.73 (1H, m) | | 3.67-3.72 (1H, m) | |
| 4'-H | 4.38 (1H, brs) | | 4.38 (1H, brs) | |
| 4'-OH | 2.00 (1H, s) | | 2.01 (1H, s) | |
| 5'-H | 1.37 (3H, brs) | | 1.38 (3H, brs) | |
| *β*-D-Chromose A |  |  |  |  |
| 1-H | 5.22-5.25 (1H, m) | | 5.22-5.28 (1H, m) | |
| 2-H_ax_ | 2.19-2.31 (1H, m) | | 2.09-2.33 (1H, m) | |
| 2-H_eq_ | 2.19-2.31 (1H, m) | | 2.09-2.33 (1H, m) | |
| 3-H | 3.99-4.21 (1H, m) | | 3.98-4.22 (1H, m) | |
| 4-H | 5.13-5.25 (1H, m) | | 5.10-5.22 (1H, m) | |
| 4-OAc | 2.19 (3H, s) | | 2.18 (3H, s) | |
| 5-H | 3.83-3.86 (1H, m) | | 3.79-3.88 (1H, m) | |
| 5-CH_3_ | 1.26 (3H, brs) | | 1.26 (3H, brs) | |
| *α*-D-Chromose B |  |  |  |  |
| 1-H | 5.12-5.14 (1H, m) | | 5.12-5.15 (1H, m) | |
| 2-H_ax_ | 1.76-1.77 (1H, m) | | 1.75-1.83 (1H, m) | |
| 2-H_eq_ | 1.76-1.77 (1H, m) | | 1.75-1.83 (1H, m) | |
| 3-H | 3.99-4.18 (1H, m) | | 3.98-4.21 (1H, m) | |
| 3-OH | 1.99 (1H, brs) | | 1.97 (1H, brs) | |
| 4-H | 3.23 (1H, brs) | | 3.24 (1H, brs) | |
| 4-OCH_3_ | 3.61 (3H, s) | | 3.61 (3H, s) | |
| 5-H | 3.81-3.90 (1H, m) | | 3.79-3.89 (1H, m) | |
| 5-CH_3_ | 1.31 (3H, brs) | | 1.34 (3H, brs) | |
| *β*-D-Chromose C |  |  |  |  |
| 1-H | 5.10-5.12 (1H, m) | | 5.10-5.12 (1H, m) | |
| 2-H_ax_ | 1.76 (1H, brs) | | 1.75 (1H, brs) | |
| 2-H_eq_ | 2.45-2.53 (1H, m) | | 2.45-2.68 (1H, m) | |
| 3-H | 3.61-3.67 (1H, m) | | 3.60-3.67 (1H, m) | |
| 4-H | 3.13-3.16 (1H, m) | | 3.13-3.16 (1H, m) | |
| 4-OH | 4.52 (1H, brs) | | 4.53 (1H, brs) | |
| 5-H | 3.25-3.40 (1H, m) | | 3.34-3.39 (1H, m) | |
| 5-CH_3_ | 1.36 (3H, brs) | | 1.34 (3H, brs) | |
| *β*-D-Chromose D |  |  |  |  |
| 1-H | 4.62-4.66 (1H, m) | | 4.60-4.66 (1H, m) | |
| 2-H_ax_ | 1.66-1.81 (1H, m) | | 1.73-1.81 (1H, m) | |
| 2-H_eq_ | 2.19-2.41 (1H, m) | | 2.17-2.35 (1H, m) | |
| 3-H | 3.53 (1H, brs) | | 3.53 (1H, brs) | |
| 4-H | 3.13-3.16 (1H, m) | | 3.13-3.16 (1H, m) | |
| 4-OH | 4.01 (1H, brs) | | 4.05 (1H, brs) | |
| 5-H | 3.27-3.43 (1H, m) | | 3.29-3.41 (1H, m) | |
| 5-CH_3_ | 1.39 (3H, brs) | | 1.33 (3H, brs) | |
| *α*-L-Chromose E |  |  |  |  |
| 1-H | 5.02-5.04 (1H, m) | | 5.02-5.05 (1H, m) | |
| 2-H_ax_ | 1.99-2.05 (1H, m) | | 2.02-2.05 (1H, m) | |
| 2-H_eq_ | 2.05 (1H, brs) | | 2.05 (1H, brs) | |
| 3-CH_3_ | 1.37 (3H, s) | | 1.35 (3H, s) | |
| 3-OH | 2.41 (1H, brs) | | 2.35 (1H, brs) | |
| 4-H | 4.62 (1H, brs) | | 4.60 (1H, brs) | |
| 4-OAc | 2.27 (3H, s) | |  |  |
| 5-H | 3.99 (1H, brs) | | 3.98 (1H, brs) | |
| 5-CH_3_ | 1.26 (3H, brs) | | 1.26 (3H, brs) | |
| 2'-CH |  |  | 2.56-2.62 (1H, m) | |
| 3' and 4'-CH_3_ |  |  | 1.13 (6H, brs) | |
